# A commentary on ‘Preventing postoperative cognitive dysfunction using anesthetic drugs in elderly patients undergoing noncardiac surgery: a systematic review and meta-analysis’

**DOI:** 10.1097/JS9.0000000000001075

**Published:** 2024-01-11

**Authors:** Jing Huang, Xin Zhu, Weijie Cao, Xinru Guo, Xue Liu

**Affiliations:** Department of Anesthesiology, The First Affiliated Hospital of Dalian Medical University, Dalian, People’s Republic of China

*Dear Editor*,

Post-operative cognitive dysfunction (POCD) is defined as an overarching term, including post-operative delirium and delayed neurocognitive recovery, and is described as a decline in neuropsychological test (NPT) performance from before to after surgery, which involves impairment of memory, concentration, and information processing^1^. Zeng *et al*.^2^ performed a systematic review and meta-analysis on patients with POCD who had undergone noncardiac surgery. It appears that the two medications with the best chance of lowering the frequency of POCD in older patients undergoing noncardiac surgery were sufentanil and dexmedetomidine.

A total of 34 trials involving 4314 elderly patients undergoing noncardiac surgery were included after searching MEDLINE, EMBASE, the Cochrane Library, and the Web of Science for randomized controlled trials comparing the various anesthetic drugs for noncardiac surgery from inception until July 2022. The results of the study showed that the incidence of POCD for each anesthetic agent was as follows: fentanyl (23.9%), midazolam (11.3%), sufentanil (6.3%), desflurane (28.3%), ketamine (15.2%), placebo (27.7%), and dexmedetomidine (12.9%). When compared to placebo, pairwise and network meta-analyses revealed that dexmedetomidine significantly reduced the incidence of POCD. Furthermore, a network meta-analysis found that dexmedetomidine significantly reduced the incidence of POCD when compared to sevoflurane.

The findings provide insight into the cumulative likelihood that anesthetic medications will lower the incidence of post-operative coronary syndrome in older patients having noncancer surgery. Based on the analysis results, the drugs sufentanil and dexmedetomidine ranked top and second in terms of lowering the incidence of POCD, with respective values of 87.4% and 81.5% for the surface under the cumulative ranking curve. In conclusion, the medications sufentanil and dexmedetomidine had the best chance of lowering the prevalence of post-operative complications related to chest surgery in older patients. However, there were still a lot of restrictions. First, ‘P’ means elderly patients whose age is greater than 60 years, according to the PICOS of the study. But Chen *et al*.’s^3^ and Mansouri *et al*.’s^4^ studies, which have been included in the meta-analysis, show that not all people are older than 60 years. Second, most meta-analyses assess the risk of bias using the Risk of Bias 2 (RoB 2) tool for randomized trials at present. Although it is not necessary, RoB 2 was used to respond to developments in understanding how bias arises in randomized trials and to address user feedback on and limitations of the original tool^5^.

This study is well-structured and addresses an important topic in the field of anesthesia. To compare the incidence of POCD for different anesthetic medications and to summarize the available evidence, this study conducted a network meta-analysis. This issue has enormous ramifications for clinical practice. According to the study’s findings, sufentanil and dexmedetomidine together had the best chance of lowering the incidence of POCD in older patients having noncardiac surgery. Sevoflurane, on the other hand, was the worst anesthetic medicine in terms of lowering the incidence of POCD in older persons undergoing noncardiac surgery. To provide more compelling evidence-based medicine and further elucidate the effectiveness of sufentanil and dexmedetomidine in lowering the incidence of post-operative anxiety disorder (POCD) in older patients undergoing noncardiac surgery, more rigorously designed, high-quality randomized controlled studies with large number of samples and strict design should be developed.

## Ethical approval

This manuscript is a comment. Don’t need ethical approval.

## Consent

This manuscript is a comment. Don’t need patient consent.

## Sources of funding

Not applicable.

## Author contribution

J.H.: study concept or design, data collection, data analysis or interpretation, and writing the paper; X.Z.: data analysis or interpretation and writing the paper; W.C. and X.G.: data collection; X.L.: study concept or design, writing, and revising the paper.

## Conflicts of interest disclosure

This manuscript is a comment without conflicts of interest.

## Research registration unique identifying number (UIN)

This manuscript is a comment. Don’t need UIN.

## Guarantor

Xue Liu.

## Data availability statement

This manuscript is a comment. Don’t need a Data availability statement. However, all the data from the current study are publicly available.

## Provenance and peer review

This manuscript is a comment without being invited.
